# Mid-Regional Pro-Adrenomedullin as a Translational Biomarker of Microcirculatory Dysfunction in Sepsis: A Prospective Observational Study

**DOI:** 10.3390/medsci14010117

**Published:** 2026-03-02

**Authors:** Rachael Cusack, Alexis Garduno, Sanja Cumpf, Pramila Reyes-Morales, Marc Leone, Alfonso Blanco Fernández, Alejandro Rodriguez, Ignacio Martin-Loeches

**Affiliations:** 1 Multidisciplinary Intensive Care Research Organisation (MICRO), Department of Intensive Care Medicine, St. James’ Hospital, D08 NHY1 Dublin, Ireland; cusackra@tcd.ie; 2 Trinity College Dublin, School of Medicine, College Green, D02 PN40 Dublin, Ireland; 3 Biochemistry Department, St. James’s Hospital, James’s Street, D08 NHY1 Dublin, Ireland; 4 Conway Institute of Biomolecular and Biomedical Research, University College Dublin, Belfield, D04 V1W8, Dublin, Ireland; 5 Department of Anaesthesiology and Intensive Care, Nord Hospital, Assistance Publique Hôpitaux Universitaires de Marseille, Aix Marseille University, 13015 Marseille, France; 6 Department of Basic Medical Sciences, Faculty of Medicine and Health Sciences, Rovira & Virgili University, 43002 Tarragona, Spain; 7 Critical Care Department, Hospital Universitari de Tarragona Joan XXIII, Mallafré Guasch 4, 43007 Tarragona, Spain; 8 IISPV (Instituto de Investigación Sanitaria Pere Virgili), 43005 Tarragona, Spain; 9 Centre for Biomedical Research Network Respiratory Diseases (CIBERES), 43005 Tarragona, Spain

**Keywords:** sepsis, MR-proADM, microcirculation, endothelial dysfunction, sublingual imaging, biomarkers, SDF microscopy, critical illness

## Abstract

**Background/Objectives**: Mid-regional pro-adrenomedullin (MR-proADM) is a biomarker of endothelial dysfunction in sepsis. Its relationship with real-time microcirculatory alterations in critically ill patients remains insufficiently characterised. **Methods**: In a prospective cohort of 59 ICU patients with sepsis, serial sublingual microcirculation assessments were performed using sidestream dark field (SDF) imaging. Serum MR-proADM concentrations were measured with BRAHMS Kryptor assays. Automated software quantified microvascular structure and flow. Associations with disease severity and outcomes were evaluated using correlation, regression, and receiver operating characteristic (ROC) analyses (ClinicalTrials.gov Identifier: NCT05357339). **Results**: Higher MR-proADM concentrations at ICU admission were modestly associated with impaired microvascular perfusion (perfused number of crossings [PNOC]: ρ = −0.32; perfused De Backer density [PDBD]: ρ = −0.32; consensus proportion of perfused vessels [CPPV]: ρ = −0.26; all *p* < 0.05). Rising MR-proADM levels over time were strongly associated with worsening perfusion (ΔPDBD: ρ = 0.52; ΔPNOC: ρ = 0.54). MR-proADM correlated with SOFA and APACHE II scores and predicted the need for renal replacement therapy (AUC = 0.799, *p* = 0.041), but not ICU length of stay or hospital mortality. **Conclusions**: MR-proADM correlates with in vivo microcirculatory dysfunction in sepsis. Its dynamic association with microvascular impairment supports its potential role as a translational biomarker for monitoring endothelial and microcirculatory failure in critically ill patients.

## 1. Introduction

Sepsis and septic shock are leading causes of morbidity and mortality in critical illness, accounting for an estimated 48.9 million global cases and 11 million deaths annually [1,2]. Despite advances in antimicrobial therapy and organ support, timely prognostication and personalised management remain critical unmet needs [3]. Sepsis arises from a dysregulated host immune response, triggering widespread endothelial injury, tissue hypoxia, and multiorgan failure [4]. A hallmark of this syndrome is microcirculatory dysfunction—marked by endothelial permeability, capillary leak, and perfusion heterogeneity—which occurs even when macrocirculatory parameters are normalised, a phenomenon termed hemodynamic incoherence [5,6,7]. Persistent microvascular impairment is strongly associated with organ failure and worse outcomes [8,9], while early recovery of capillary perfusion correlates with improved prognosis [10]. Although tools such as serum lactate and central venous oxygen saturation (ScvO_2_) are used as indirect surrogates, sublingual sidestream dark field (SDF) imaging remains the gold-standard for real-time, direct microcirculatory assessment [11].

Among circulating biomarkers, mid-regional pro-adrenomedullin (MR-proADM) has emerged as a promising candidate linking systemic inflammation, endothelial stress, and vascular tone [12,13]. Derived from the more transient vasodilator adrenomedullin (ADM), MR-proADM is a plasma-stable fragment that reflects ADM’s biological activity, including endothelial barrier preservation and vasoregulation via cyclic AMP pathways [14,15,16]. Elevated serum MR-proADM concentrations have been consistently associated with sepsis severity, vasopressor requirement, and mortality, outperforming traditional inflammatory markers such as CRP and procalcitonin in some prognostic models [17,18,19]. Mechanistically, MR-proADM appears to reflect a compensatory response to endothelial dysfunction and tissue hypoperfusion [20]. However, while its association with systemic severity scores and macro-hemodynamic outcomes is well documented, its relationship with actual microcirculatory performance remains poorly defined. Whether MR-proADM trends can mirror dynamic changes in capillary perfusion and serve as a biomarker of perfusion failure remains an open and clinically important question. Precision medicine in critical care medicine will identify patient phenotypes, such as those more or less likely to respond to treatments. In this regard, MR-proADM may discern patients for whom microcirculation dysfunction is the cause of their shock.

We hypothesised that serum MR-proADM concentrations reflect sublingual microcirculatory dysfunction in patients with sepsis and septic shock. Specifically, we proposed that higher serum MR-proADM concentrations would correlate with impaired capillary perfusion, including reduced vessel density and proportion of perfused vessels. The primary objective of our study was to assess the association between serum MR-proADM concentrations and direct SDF-derived microcirculatory variables in fluid-responsive intensive care unit (ICU) patients with sepsis. Secondary objectives included evaluating MR-proADM’s correlations with inflammatory biomarkers (c-reactive protein (CRP), lactate, white blood cell count (WBC)) and clinical severity scores (sequential organ failure assessment (SOFA), acute physiology and chronic health evaluation (APACHE)).

## 2. Materials and Methods

### 2.1. Definitions

Sepsis and septic shock were defined according to Sepsis-3 criteria [21]. Fluid responsiveness was assessed using dynamic indices, including pulse pressure variation and passive leg raise. Bacteraemia patients were those with microbiologically confirmed infection managed outside the ICU.

### 2.2. Study Design and Setting

This prospective observational study was conducted in the mixed medical–surgical intensive care unit (ICU) of St James’s Hospital, a tertiary referral centre in Dublin, Ireland. The ICU comprises 35 beds and admits over 2000 patients annually. The study integrates data from two prospective cohorts collected between September 2021 and May 2023.

The first cohort included patients with microbiologically confirmed infection and sepsis or bacteraemia who had serum sampling for MR-proADM and clinical data recorded. The second cohort was embedded within the MICALB21 microcirculation study (ClinicalTrials.gov Identifier: NCT05357339) and focused on critically ill patients with sepsis undergoing serial sublingual microcirculatory assessment [22]. All patients were followed until ICU, hospital discharge or death.

The study was conducted in accordance with the Declaration of Helsinki and was approved by the joint board of St James Hospital and Tallaght University Hospital research ethics committee (SJH/TUH REC). The approval number granted to this study is 2019-11-269040 5642 on the 26th of April 2021. The consent process was developed in accordance with local guidelines and under the supervision of the Health Research Consent Declaration Committee (HRCDC), reference identification number 20-031-AF1. An annual review of consent processes and adherence to procedure and guidelines was undertaken.

### 2.3. Participants

For the MICALB21 cohort, inclusion criteria were: (i) age ≥ 18 years; (ii) sepsis or septic shock defined according to Sepsis-3 criteria; (iii) ICU admission; and (iv) fluid responsiveness defined by pulse pressure variation (PPV) > 13%, with subsequent fluid resuscitation until no longer responsive. To measure patients’ fluid responsiveness, PPV was measured with tidal volumes set to 8–10 mL/kg for the duration of the intervention and measurement. Patients were then returned to lung protective ventilation settings (or previous mechanical ventilation settings). These patients underwent protocolised microcirculatory imaging.

Exclusion criteria included pregnancy, expected death within 24 h, inability to obtain informed consent, and recent oral or sublingual surgery precluding imaging. Patients not mechanically ventilated were excluded, as they could not have their fluid responsiveness measured using PPV.

Patients included only for MR-proADM measurement and clinical correlation were eligible if they were ≥18 years old and had microbiologically confirmed infection; ICU admission was not required for this subgroup.

### 2.4. Outcomes

The primary outcome was the association between serum MR-proADM concentrations and real-time sublingual microcirculatory perfusion assessed by sidestream dark field (SDF) imaging. Key microcirculatory variables were the perfused number of crossings (PNOC), perfused De Backer density (PDBD), and consensus proportion of perfused vessels (CPPV), selected as robust indicators of capillary flow and functional microvascular density. Patients in the MICALB21 cohort, mechanically ventilated in ICU, were included in this analysis.

Secondary outcomes included associations between MR-proADM and inflammatory markers (C-reactive protein [CRP], lactate, white cell count), severity scores (SOFA and APACHE II), and clinical endpoints including vasopressor use, Acute Kidney Injury Network (AKIN) scores, creatinine, renal replacement therapy (RRT), ICU survival, and length of stay. Patients in the second cohort (bacteraemia without ICU admission) were included in this analysis.

### 2.5. Microcirculation Assessment

Sublingual microcirculation was assessed using the Microscan SDF camera (Microvision Medical, Amsterdam, The Netherlands), which uses green light (540 nm) to visualise flowing erythrocytes. Imaging was performed at three timepoints: ICU admission (T1), approximately 60 min after fluid resuscitation and haemodynamic stabilisation (T2), and 24 h later (T3).

At each timepoint, five high-quality video sequences were recorded following removal of saliva and analysed using AVA 4.3 software in accordance with international consensus guidelines. Microcirculatory variables included vessel density and perfusion indices for total and small vessels (<20 μm), including PNOC, PDBD, CPPV, and their small-vessel equivalents.

### 2.6. Biomarker Measurement

Blood samples were collected in citrate tubes, centrifuged immediately, and stored at −80 °C. Serum MR-proADM concentrations were measured in batch using the B.R.A.H.M.S. Kryptor GOLD platform (Thermo Fisher Scientific, Merelbeke, Belgium), employing TRACE technology for quantitative detection of the stable mid-regional fragment of adrenomedullin. Serum biomarkers, including MR-proADM, were measured at three timepoints: time 0 (diagnosis/confirmation of bacteraemia or sepsis), time 2 (24 h following first sample) and time 3 (3–5 days after initial diagnosis or first sample). Patients who did not survive to time 3 could not be sampled.

MR-proADM serum concentrations at time 0 were analysed with microcirculation images at T1; time 2 MR-proADM was analysed with microcirculation images obtained at T2; patients alive at time 3 had MR-proADM and microcirculation images from day 3–5 analysed together.

### 2.7. Statistical Analysis

Statistical analyses were performed using R (version 4.3.1). Spearman correlation was used to assess associations between MR-proADM concentrations and microcirculatory, biochemical, and clinical variables. Linear regression explored continuous relationships. Temporal associations were examined using delta analyses comparing changes in MR-proADM with changes in microcirculatory indices between timepoints. Logistic regression and receiver operating characteristic (ROC) analyses evaluated the predictive value of MR-proADM for binary outcomes. Missing data were handled by pairwise exclusion. Statistical significance was defined as *p* < 0.05.

## 3. Results

### 3.1. Clinical Characteristics

**Figure 1:**
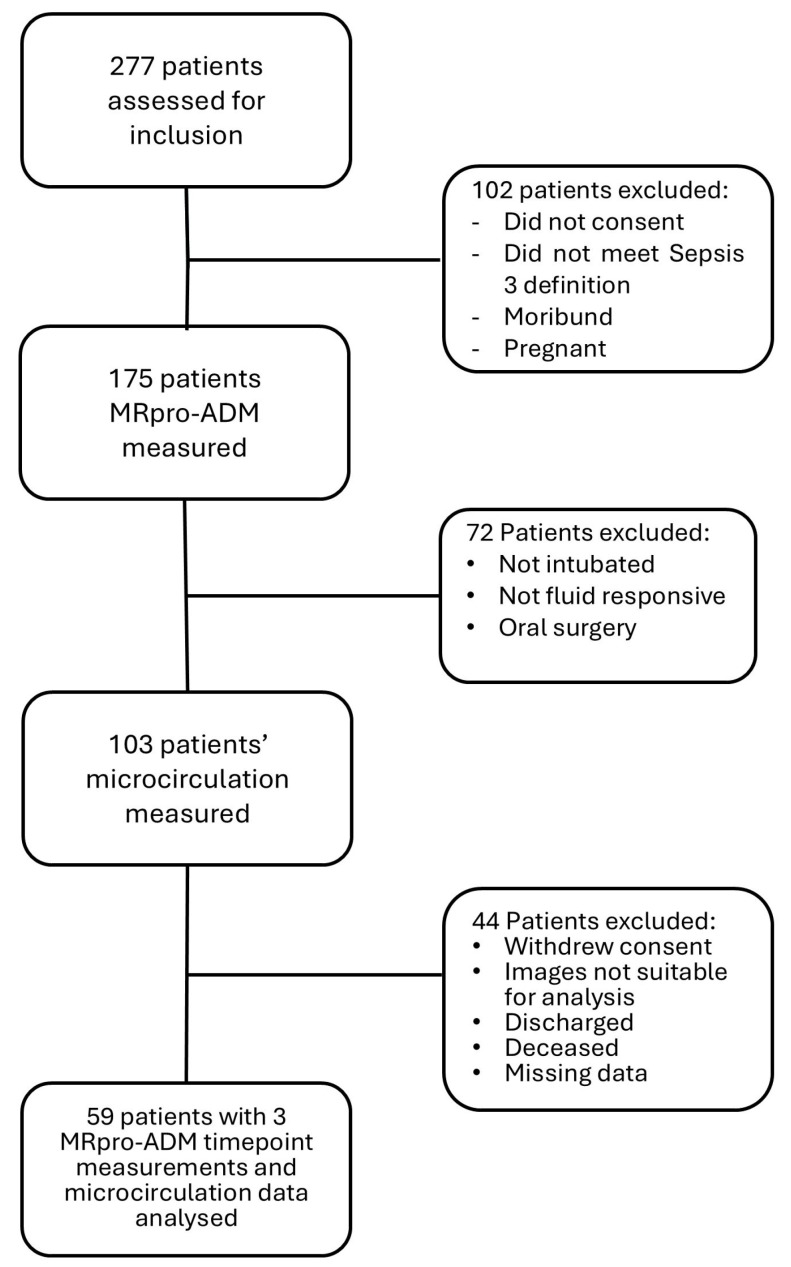
Flowchart depicting the inclusion of patients in study analysis. This study was nested within the MICALB21 study, and patients who had microcirculation data collected were intubated and ventilated for fluid responsiveness PPV measurements. Patients who were discharged or deceased before they had all their timepoints measured were excluded from final analysis.

A total of 103 patients with sepsis or septic shock underwent sublingual microcirculatory assessment, of whom 59 had paired baseline serum MR-proADM measurements and were included in the primary analysis (Figure 1). Forty-seven patients had repeat MR-proADM sampling at 24 h.

The cohort had a mean age of 54 years (range 19–84), and 60% were male. Illness severity was high, with a mean APACHE II score of 27 (range 7–43) and a mean SOFA score of 9.5 (range 4–20). The median ICU length of stay was 24 days (range 1–190) and hospital length of stay was 53 days (range 3–242). ICU survival to discharge was 70.3%. Baseline demographic, biochemical, and clinical characteristics are summarised in Table 1.

#### 3.1.1. Association Between MR-proADM and Microcirculatory Perfusion

**Figure 2:**
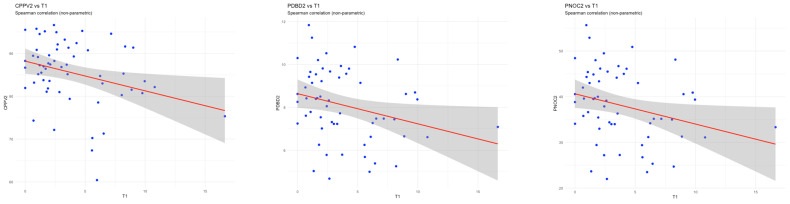
Scatter plots with linear regression lines (red) and 95% confidence intervals (grey shading) demonstrating the relationship between MR-proADM concentrations at presentation (MR-proADM time 0 and microcirculation T1) and sublingual microcirculatory parameters for patients in the MICALB21 cohort. Significant inverse associations were observed between MR-proADM and Consensus PPV (% perfused vessels), De Backer density, and proportion of perfused vessels (small vessels), indicating that higher MR-proADM levels were associated with impaired microvascular density and perfusion.

At ICU admission, higher serum MR-proADM concentrations were significantly associated with impaired sublingual microcirculatory perfusion. Specifically, MR-proADM correlated inversely with the perfused number of crossings (PNOC; ρ = −0.32, *p* = 0.014), perfused De Backer density (PDBD; ρ = −0.32, *p* = 0.015), and consensus proportion of perfused vessels (CPPV; ρ = −0.26, *p* = 0.045) (Figure 2).

Admission MR-proADM also predicted persistent microcirculatory dysfunction following initial haemodynamic resuscitation. Stronger inverse associations were observed at 60 min post-resuscitation (T2) for PNOC (ρ = −0.32, *p* = 0.017) and PDBD (ρ = −0.33, *p* = 0.016), indicating limited microvascular recruitment despite macro-haemodynamic optimisation.

**Figure 3:**
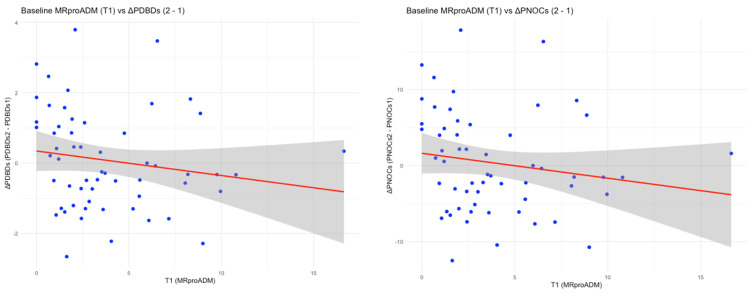
Linear regression scatterplots showing the change in PNOC and PDBD from baseline to one hour correlated to the first measurement of serum MR-proADM, PNOC = perfused number of crossings (r = −0.27, *p* = 0.0409), PDBD = perfused De Backer density (r = −0.27, *p* = 0.041). MICALB21 cohort of patients. Red line indicates regression trend, with blue dots representing timepoint MR-proADM measurements. X-axis indicates MR-proADM serum concentration at T1 and Y-axis represents the change in perfused De Backer Density and perfused number of crossings from pre-resuscitation to post-resuscitation.

Baseline MR-proADM was further associated with attenuated early microcirculatory response to fluid resuscitation. Higher MR-proADM concentrations correlated with smaller improvements in PNOC and PDBD during the first hour (ΔPNOC and ΔPDBD; both ρ = −0.27, *p* < 0.05) (Figure 3).

**Figure 4:**
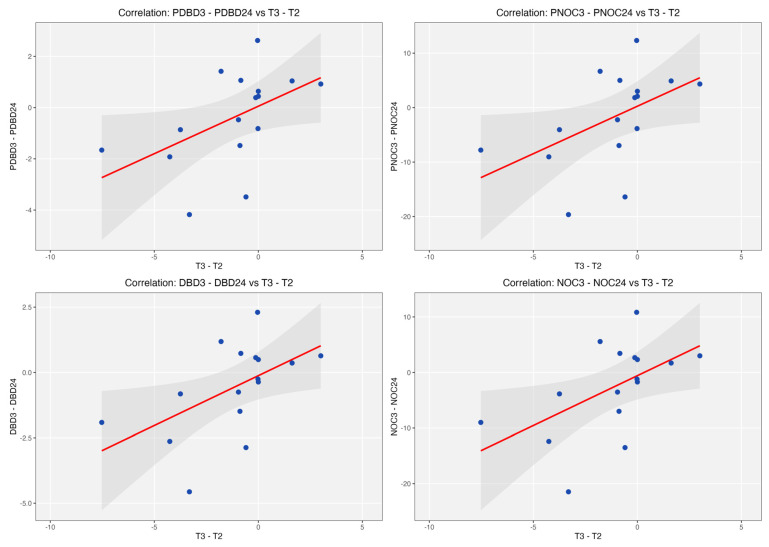
Linear regression scatterplots showing how the change in microcirculation variables correlates to the change in MR-proADM. PNOC = perfused number of crossings (ρ = 0.54, *p* = 0.030); PDBD = perfused De Backer density (ρ = 0.54, *p* = 0.030); NOC = number of crossings (ρ = 0.52, *p* = 0.039); DBD = DeBacker density (ρ = 0.52, *p* = 0.039). MICALB21 cohort of patients. Red line represents linear regression. X-axis represents change in MR-proADM from 24hours to day 3-5 (T2 to T3) and y-axis represents change in perfused number of crossings, number of crossings, perfused De Backer density and De Backer density of microcirculation from 24 hours to day 3-5. There were notably fewer patients with paired results at these time points, making the results difficult to interpret.

In longitudinal analyses, increases in serum MR-proADM between 24 h and days 3–5 were associated with worsening microcirculatory perfusion, including reductions in PNOC and PDBD (ρ = 0.54, *p* = 0.03 for both), as well as total vessel density metrics (Figure 4). However, missing data at later timepoints limits the strength of these findings.

No significant association was observed between immediate post-resuscitation microcirculatory indices and subsequent 24 h changes in MR-proADM, highlighting a temporal dissociation between rapid microvascular responses and slower biomarker kinetics.

#### 3.1.2. MR-proADM and Inflammatory Burden

**Figure 5:**
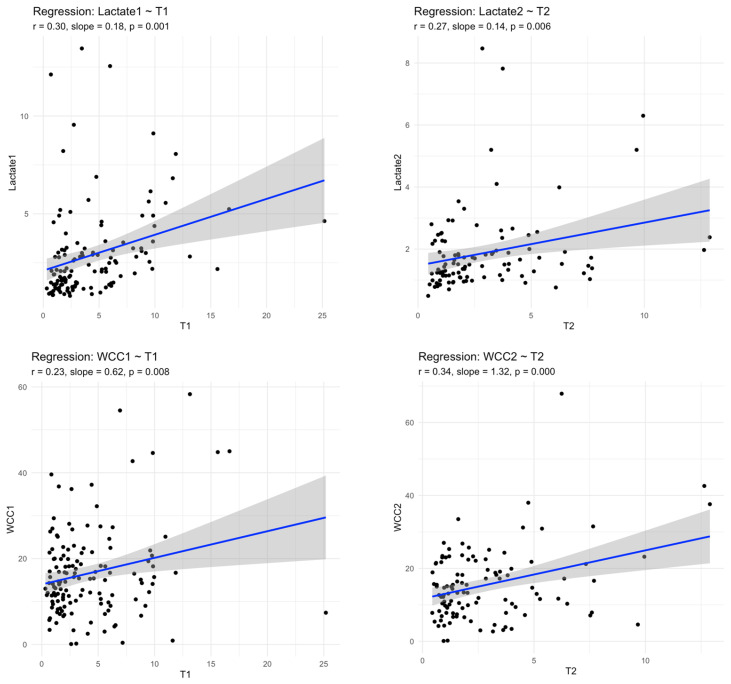

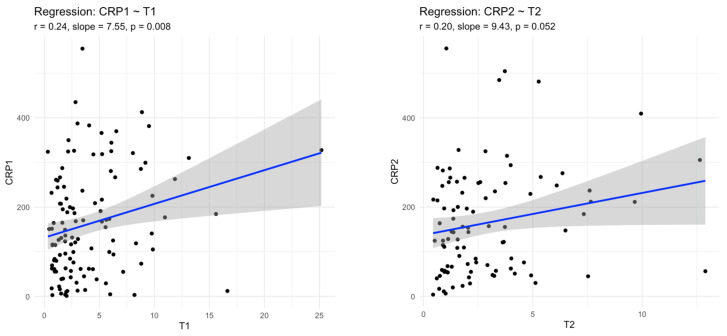
Linear regression plots showing the association between MR-proADM concentrations and clinical/biochemical markers at presentation (T1) and 24 h (T2). Scatter plots display individual patient values with fitted regression lines (blue) and 95% confidence intervals (grey shading). Significant positive correlations were observed between MR-proADM and lactate at T1 (r = 0.30, *p* = 0.001) and T2 (r = 0.27, *p* = 0.006), white blood cell count (WBC) at T1 (r = 0.23, *p* = 0.008) and T2 (r = 0.34, *p* < 0.001), and C-reactive protein (CRP) at T1 (r = 0.24, *p* = 0.008). A borderline association was seen between MR-proADM and CRP at T2 (r = 0.20, *p* = 0.052). MICALB21 and bacteraemia cohorts.

Serum MR-proADM concentrations were positively correlated with established inflammatory and metabolic markers. At admission, MR-proADM correlated with serum lactate (ρ = 0.30, *p* < 0.001), white cell count (ρ = 0.23, *p* = 0.008), and C-reactive protein (ρ = 0.24, *p* = 0.008). These associations persisted at 24 h, particularly for lactate and white cell count (Figure 5).

#### 3.1.3. MR-proADM and Severity

**Figure 6:**
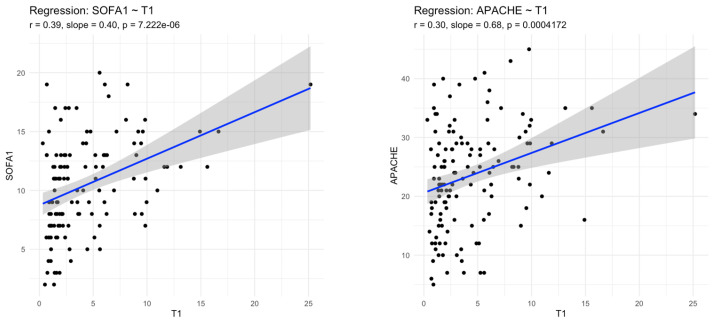
Associations between clinical severity scores and MR-proADM serum concentrations in patients on admission to ICU.

MR-proADM concentrations increased stepwise with clinical severity, with the lowest levels observed in bacteraemia without sepsis and the highest levels in septic shock (ANOVA *p* < 0.001). Admission MR-proADM correlated significantly with SOFA (ρ = 0.39, *p* < 0.001) and APACHE II scores (ρ = 0.30, *p* < 0.001) (Figure 6).

#### 3.1.4. Clinical Outcomes

Admission MR-proADM was not significantly associated with ICU survival, prolonged ICU stay (>28 days), or hospital length of stay.

#### 3.1.5. Acute Kidney Injury and Renal Replacement Therapy

**Figure 7:**
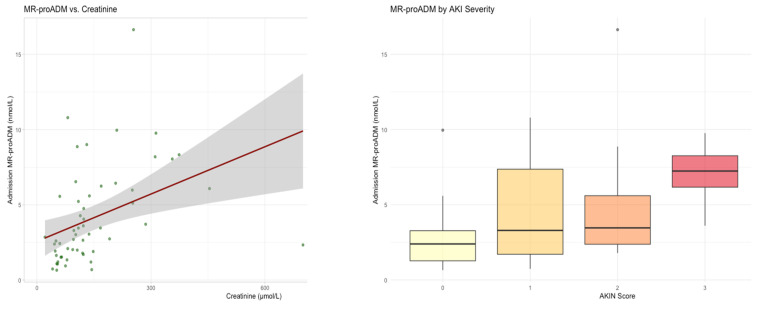
Linear regression between MR-proADM and serum creatinine, Spearman ρ = 0.576, *p* = <0.001 and boxplots of KDIGO AKIN Score, by MR-proADM serum concentration, Spearman ρ = 0.455, *p* = 0.0005. x-axis represents KDIGO AKIN grades of kidney injury 0-3, light yellow = 0, dark yellow = 1, orange = 2, red = 3.

**Figure 8:**
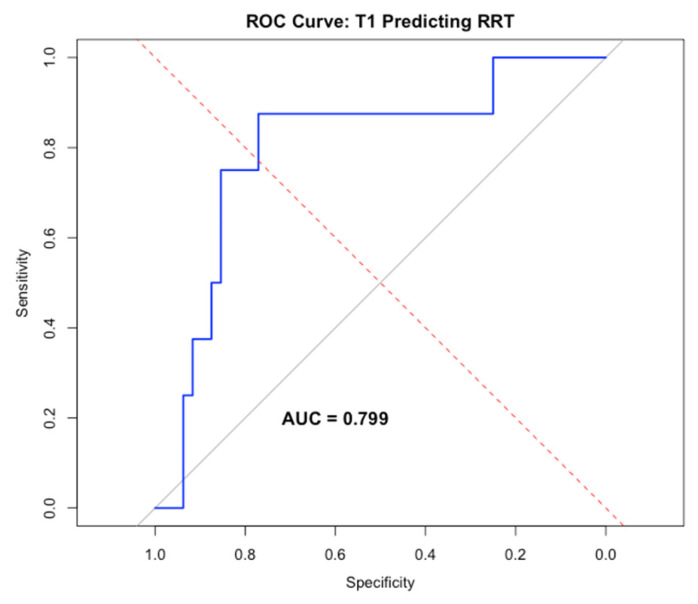
ROC curve with AUC 0.799 showing MR-proADM level is associated with the need for renal replacement therapy and logistic regression curve.

MR-proADM demonstrated a strong positive association with renal dysfunction. Admission MR-proADM correlated with serum creatinine (ρ = 0.58, *p* < 0.001) and increased progressively with worsening KDIGO acute kidney injury stage (Figure 7). Higher MR-proADM concentrations were independently associated with the need for renal replacement therapy (β = 0.25, *p* = 0.041), with good discriminative performance (AUC = 0.799) (Figure 8).

## 4. Discussion

In this prospective observational study, we examined the relationship between serum mid-regional pro-adrenomedullin (MR-proADM) concentrations and real-time microcirculatory function in critically ill patients with sepsis. Our principal finding is that higher MR-proADM concentrations at ICU admission are associated with impaired sublingual microvascular perfusion, characterised by reduced capillary density and a lower proportion of perfused vessels. Importantly, elevated MR-proADM identified a subgroup of patients whose microcirculation failed to improve despite early macrocirculatory resuscitation. These findings support the concept that MR-proADM reflects endothelial and microvascular dysfunction rather than macrohaemodynamic instability alone.

A key observation was the inverse association between baseline MR-proADM concentration and early microcirculatory recruitment following fluid resuscitation. Patients with higher MR-proADM levels demonstrated attenuated improvements—or even deterioration—in consensus proportion of perfused vessels (CPPV), perfused number of crossings (PNOC), and perfused De Backer density (PDBD) within the first 60 min. This suggests that MR-proADM identifies a phenotype of “fixed” or refractory microcirculatory dysfunction, in which restoration of systemic blood pressure and flow does not translate into effective capillary perfusion. In this context, MR-proADM appears to capture a dimension of shock severity that is invisible to conventional haemodynamic targets.

Notably, MR-proADM at admission predicted microcirculatory function after initial resuscitation (T2) more robustly than at baseline (T1). This temporal relationship reinforces the concept that MR-proADM identifies patients in whom microvascular dysfunction persists despite macrocirculatory optimisation. Such patients may represent a biologically distinct subgroup characterised by endothelial barrier failure, glycocalyx disruption, and impaired vasoreactivity. These findings align with growing evidence that persistent microcirculatory alterations—rather than hypotension per se—are strongly linked to organ failure and adverse outcomes in sepsis.

Despite these associations, our data do not support MR-proADM as a direct surrogate marker of microcirculatory failure. While correlations were statistically significant, effect sizes were modest, and not all microcirculatory variables demonstrated consistent associations. Structural indices such as small-vessel counts and crossings were less strongly linked to MR-proADM concentration, likely reflecting the complex and heterogeneous determinants of microvascular architecture in critically ill patients. These include inter-individual differences in comorbidity burden, regional heterogeneity of perfusion, timing and type of resuscitation, and ongoing inflammatory injury.

Beyond the microcirculation, MR-proADM stratified patients across a clear severity spectrum, from bacteraemia without sepsis to septic shock, consistent with the prior literature. Its strong correlation with SOFA and APACHE II scores reinforces its role as an integrative marker of disease severity. Associations with CRP, serum lactate, and leukocytosis further support MR-proADM as a marker of systemic inflammation and metabolic stress. These findings corroborate existing evidence positioning MR-proADM as an indicator of global disease burden in sepsis.

We also observed strong associations between MR-proADM and renal dysfunction. Serum MR-proADM concentrations increased progressively with worsening acute kidney injury and were independently associated with the requirement for renal replacement therapy. This relationship likely reflects a combination of reduced renal clearance and heightened endothelial injury in more severe shock states. Importantly, the correlation between MR-proADM and microcirculatory “failure-to-improve” may therefore partially reflect the presence of concomitant renal dysfunction and advanced endothelial damage. These interdependencies highlight the difficulty of disentangling causality in complex, multi-organ failure syndromes such as sepsis.

From a mechanistic standpoint, our findings are biologically plausible. MR-proADM is a stable surrogate of adrenomedullin, a peptide with potent vasodilatory, anti-inflammatory, and endothelial barrier-regulating properties [23]. Adrenomedullin is upregulated in response to inflammatory cytokines and endothelial stress and plays a central role in sepsis-induced vasoplegia and capillary leak. Endothelial dysfunction is a defining feature of septic shock, contributing directly to tissue hypoxia, impaired oxygen extraction, and multi-organ failure [24,25]. By linking MR-proADM concentrations to direct visualisation of microvascular perfusion, our study strengthens the conceptual bridge between molecular endothelial injury and functional microcirculatory failure.

Previous studies have demonstrated that MR-proADM clearance over time is associated with clinical improvement and reductions in SOFA scores [26]. Our data extend these observations by showing that biomarker dynamics and microcirculatory recovery do not necessarily occur in parallel. We observed a temporal dissociation between early microcirculatory responses to resuscitation and subsequent changes in serum MR-proADM concentration. While MR-proADM at admission identified patients with persistent microcirculatory dysfunction after 60 min, changes in MR-proADM over the following 24 h did not correlate with immediate improvements in microvascular perfusion. This suggests that MR-proADM reflects a broader and slower-resolving state of endothelial and cellular stress, rather than acute capillary recruitment alone.

This dissociation has important clinical implications. The microcirculation can respond—or fail to respond—within minutes to fluids and vasopressors at the bedside, whereas MR-proADM clearance likely reflects longer-term processes such as endothelial repair, resolution of inflammation, or ongoing cellular injury. Baseline MR-proADM may therefore be best interpreted as a marker of underlying microcirculation-driven shock, identifying patients less likely to respond to early resuscitation. Longitudinal biomarker trajectories may then represent a distinct phase of disease biology, reflecting either endothelial recovery or persistent injury.

The lack of association between certain microcirculatory parameters and MR-proADM also warrants consideration. Microcirculatory assessment is inherently subject to regional heterogeneity and sampling variability, even when performed according to international consensus standards. It is possible that localised perfusion abnormalities were not fully captured by sublingual imaging, or that inter-observer and technical variability diluted specific associations. Moreover, the expression and clearance of MR-proADM may be influenced by factors independent of microvascular perfusion, including neurohumoral activation, renal function, and systemic inflammatory load.

Several limitations must be acknowledged. This was a single-centre, observational study using a convenience sample, limiting causal inference. The modest sample size restricted subgroup analyses and adjustment for potential confounders such as timing of vasopressor initiation, fluid balance beyond 24 h, and source-specific sepsis phenotypes. Missing MR-proADM samples—particularly at later timepoints—were unavoidable due to the requirement for immediate centrifugation, limiting out-of-hours collection. Although microcirculatory imaging was performed by a single trained operator and analysed using semi-automated software to minimise bias, operator dependency and sampling error remain inherent limitations of SDF imaging.

Despite these constraints, the study population reflects a real-world, heterogeneous ICU cohort, enhancing the external relevance of our findings. Importantly, our results should be interpreted as hypothesis-generating rather than definitive. Larger, multicentre studies with standardised microcirculatory protocols and more frequent biomarker sampling are required to validate these observations and to explore whether MR-proADM-guided stratification can inform targeted therapeutic strategies.

Taken together, our findings position MR-proADM as a potential molecular link between endothelial disruption and microvascular dysfunction in sepsis. By aligning with both direct bedside imaging and systemic indicators of severity, MR-proADM offers a unique opportunity to integrate molecular and physiological assessments. As critical care moves toward precision-based approaches, combining biomarkers with functional microcirculatory assessment may allow earlier identification of patients at risk of refractory shock and guide personalised resuscitation strategies.

## 5. Conclusions

This study demonstrates that MR-proADM is associated with impaired sublingual microcirculatory perfusion and increased disease severity in sepsis. Elevated MR-proADM identifies patients with persistent microcirculatory dysfunction despite early macrocirculatory resuscitation, supporting its role as a marker of endothelial stress rather than haemodynamic instability alone. While not a surrogate for microcirculatory failure, MR-proADM bridges molecular pathology and tissue-level perfusion abnormalities and holds promise for risk stratification and monitoring of disease trajectory. Further longitudinal and multicentre studies are required to define its role within precision-based sepsis management.

## Figures and Tables

**Table 1:** Demographic statistics of the cohort. Times 1, 2 and 3 refer to MR-proADM serum concentrations at those respective timepoints, described above. LOS: length of stay, ICU: intensive care unit, WBC: white blood cell count, CRP: c-reactive protein. 1, 2 refer to timepoints when samples were collected—time 1 diagnosis/ICU admission, time 2—24 h later. All patients recruited in ICU (n = 59) were on noradrenaline at time 1 and 84% of the cohort (n = 39) were receiving noradrenaline at time 2, 24 h timepoint.

	Mean (Min–Max)
Age	54 (19–84)
Sex (m)	60%
MR-proADM concentration at Time 1 (nmol/L)	3.02 (0.65–16.64)
MR-proADM concentration at Time 2 (nmol/L)	2.55 (0.45–9.95)
MR-proADM concentration at Time 3 (nmol/L)	1.86 (0.49–11.57)
APACHE	27 (range 7–43)
SOFA	9.5 (4–20)
ICU LOS	24 days (1–190),
LOS	53 days (3–242).
ICU Survival to discharge	70.3%
Lactate at Time 1 (mmol/L)	2.6 (0.6–13.5)
Lactate at Time 2 (mmol/L)	1.9 (0.49–8.5)
WBC count at Time 1 (×10^9^/L)	16.18 (0.1–86.4)
WBC count at Time 2 (×10^9^/L)	15.17 (0.1–67.9)
CRP at Time 1 (mg/dL)	145.09 (1.21–555)
CRP at Time 2 (mg/dL)	145.31 (1.53–555.62)
Noradrenaline tartrate dose (mcg/kg/min) Time 1	0.225 (0.04–0.812)
Noradrenaline tartrate dose (mcg/kg/min) Time 2	0.115 (0–0.74)
Fluid balance at 24 h (mL)	2633 (−967–8863)
Respiratory sepsis (% of patient cohort)	52%
Abdominal sepsis (% of patient cohort)	34%
Miscellaneous (urosepsis/cellulitis/osteomyelitis)	14%

## Data Availability

The original contributions presented in this study are included in the article. Further inquiries can be directed to the corresponding author.
